# Family factors to predict adolescents’ emotional health by decision tree model: A comparison between normally developed group and chronic-condition group

**DOI:** 10.3389/fpubh.2023.1087547

**Published:** 2023-03-16

**Authors:** Yi Huang

**Affiliations:** ^1^Department of Psychology, Faculty of Social Studies, Masaryk University, Brno, Czechia; ^2^Psychology Research Institute, Faculty of Social Studies, Masaryk University, Brno, Czechia

**Keywords:** adolescent, family environment, emotional health, chronic condition, decision tree

## Abstract

The increasing trend of adolescents’ emotional symptoms has become a global public health problem. Especially, adolescents with chronic diseases or disabilities face more risks of emotional problems. Ample evidence showed family environment associates with adolescents’ emotional health. However, the categories of family-related factors that most strongly influence adolescents’ emotional health remained unclear. Additionally, it was not known that whether family environment influences emotional health differently between normally developed adolescents and those with chronic condition(s). Health Behaviours in School-aged Children (HBSC) database provides mass data about adolescents’ self-reported health and social environmental backgrounds, which offers opportunities to apply data-driven approaches to determine critical family environmental factors that influence adolescents’ health. Thus, based on the national HBSC data in the Czech Republic collected from 2017 to 2018, the current study adopted a data-driven method, classification-regression-decision-tree analysis, to investigate the impacts of family environmental factors, including demographic factors and psycho-social factors on adolescents’ emotional health. The results suggested that family psycho-social functions played a significant role in maintaining adolescents’ emotional health. Both normally developed adolescents and chronic-condition(s) adolescents benefited from communication with parents, family support, and parental monitoring. Besides, for adolescents with chronic condition(s), school-related parental support was also meaningful for decreasing emotional problems. In conclusion, the findings suggest the necessity of interventions to strengthen family-school communication and cooperation to improve chronic-disease adolescents’ mental health. The interventions aiming to improve parent-adolescent communication, parental monitoring, and family support are essential for all adolescents.

## Introduction

Adolescence is a peak stage for the onset of emotional problems (1–4). Moreover, the increasing trend of adolescents’ emotional problems has been noted across many regions. For instance, compared to 1986, the prevalence of adolescents’ emotional problems in 2006 was twice higher in England (1). A systematic review suggested that adolescents’ emotional health problems rise to a critical global public health issue, and especially the prevalence is remarkable in high-income countries (4). Besides, emotional symptoms hinder adolescents’ social development and academic performance (5–7).

Totsika and Sylva’s theory suggested that due to youth’s basic developmental needs, the family environment can influence the next generation’s emotional health from two aspects: family demographic background (e.g., family income and parental employment) and family psycho-social function (e.g., emotional warmth and parental guidance) (8). There were studies echoing the theory. A Danish longitudinal study compared the differences in adolescents’ emotional symptoms between low, medium, and high socioeconomic status (SES) families, and it found that adolescents from medium and high SES families face less risk of emotional symptoms than those from low SES families. However, the increasing trend of adolescents’ emotional symptoms crossing the years is most notable in high-SES families (9). A meta-analysis work noted a significant correlation between SES and children’s and adolescents’ affective disorders (10). In addition, some evidence suggested the importance of family psychological support for adolescents’ affective development. A review from the clinical empirical perspective summarized that, compared to the individual psychological treatment, it is more effective to treat adolescents’ depression by including other family members’ participation and enhancing family communications and supports (11).

It is worth noting that compared to normally developed adolescents, adolescents with chronic disease or disabilities face more challenges for emotional health (12, 13). Therefore, on the basis of Totsika and Sylva’s theory, they need more psychological support from the family (8). A previous study proved that for adolescents with chronic disease, parental involvement and support have positive impacts on their disease self-management, life quality, and wellbeing (14).

Nonetheless, to the best of my knowledge, there was no study to probe whether family environmental influencers differently affect the emotional health between normally developed adolescents and adolescents with chronic clinical condition(s). Furthermore, the order of importance of family factors for adolescents’ emotional health remained unclear. World Health Organization initiated a cross-national project named Health Behaviours in School-aged Children (HBSC) to investigate adolescents’ health and the background information, which provides mass data and multi variables.^1^ Data-driven methods (e.g., decision tree model and Bayesian network analysis) efficiently help to select the strongest predictors from all candidate independent variables. Thus, it has a great potential to adopt data-driven approaches to examine the associations between various possible predictors and the target outcome based on big databases like HBSC. However, a systematic scoping review suggested even though HBSC database provides opportunities for researchers to use data-driven approaches, very few studies used the methods (15). For filling the gap in previous research studies, the current study aimed to adopt HBSC database and decision tree models to explore the most important family environmental influencers that affect emotional health among adolescents with or without chronic condition(s).

## Methods

### Data resource

This study adopted the HBSC data collected in the Czech Republic from the year 2017 to 2018.

A two-stage sampling method was used to collect a representative national sample. In the first stage, 227 schools nationwide were randomly selected, and in the second stage, each school decided on one class in 11/13/15-year-old grades to participate in, respectively. Students answered questionnaires voluntarily. A total of 13,377 effective responses were collected (16). 1,436 participants did not report their medical diagnosis of chronic diseases or disabilities. Thus, only 11,941 observations were included in the analysis. Among them, 3,067 adolescents were diagnosed with long-term illnesses or disabilities and the left 8,874 adolescents were without chronic conditions. The current study included all the family-related factors in the HBSC database, which were introduced in the measurement section subsequently.

### Measurements

*Emotional health* was measured by three items in HBSC Symptoms Checklist. Participants were required to rate the frequency of three symptoms from 1 (“about everyday”) to 5 (“rarely or never”), including feeling low, irritable, and nervous. The McDonald’s omega values were 0.73 and 0.72 for the normally developed group and the chronic-condition(s) group, respectively, which indicated an acceptable internal consistency for the two groups.

*Family affluence scale (FAS)* assessed family material affluence, which asked a series of family material sets or activities, including cars, computers, bathrooms, dishwasher, family holidays, and adolescents’ individual bathrooms. FAS is a good SES indicator (17). The responses of the items were not in the same range. Furthermore, the variable types also differed. For instance, the first item in the scale asked the number of cars, and the matched response was an order variable from 1 (“no”) to 3 (“yes, two or more”). However, the reaction of the second item was a categorical variable. Participants were required to answer if they had their own bedroom by “yes” or “no.” Therefore, the current study did not calculate alpha or McDonald’s omega value to investigate the internal consistency index.

*Subjective perception of family wealth* was asked by a single 5-point (from “very well off” to “not at all well off”) item “How well off do you think your family is?.” I reversed the item scores.

*Talks to parents* was assessed by the question “How easy is it for you to talk to your mother/father about things that really bother you?.” Participants responded from 1 (“very easy”) to 5 (“do not have or see”). The scores were reversed.

*Parental monitoring* was measured by twelve 4-point items, among which six items focused on mothers and the other six items were on fathers. Adolescents responded from “she/he knows a lot” to “do not have or do not see mother/father.” A series of questions were about if parents really knew about adolescents’ social activities, for example, “mother/father knows my friends” and “mother/father knows how I spend money.” The reliability was good for the normally developed group (McDonald’s omega = 0.90) and the chronic group (McDonald’s omega = 0.91). I reversed the scores.

*School-related parental support* was measured by five items rated from 1 (“strongly agree”) to 5 (“strongly disagree”). The items asked parental support in terms of adolescents’ school activities, for instance, “my parents are interested in what happens to me at school” and “my parents encourage me to do well at school.” The internal consistency was good for the normally developed group (McDonald’s omega = 0.87) and the chronic group (McDonald’s omega = 0.88). Participants’ scores were reversed.

*The family subscale of the multidimensional scale of perceived social support* was adopted to measure family support. The instrument needed adolescents to answer four aspects from 1 (“very strongly disagree”) to 7 (“very strongly agree”): help, emotional support, problem talking, and help of decision making. The McDonald’s omega was 0.98 among normally developed adolescents and it was 0.97 in the chronic group.

*Family activities* were measured by a 5-point scale. It comprised 9 items and required participants to report the frequency of the following family activities from “everyday” to “never”: watching TV/video, playing indoor games, playing computer games, eating a meal, going for a walk, going places, visiting friends or relatives, playing sports, and sitting and talking about things. The scores were reversed. The reliability was good in the chronic group (McDonald’s omega = 0.85) and the normally developed group (McDonald’s omega = 0.84).

### Data analysis

The current study adopted the Classification and Regression Decision Tree (CRT) analysis, a data-driven method. Unlike the traditional hypothesis-driven approaches, which raise prior assumptions based on previous theories, the data-driven methods do not make initial assumptions. Moreover, data-driven approaches efficiently select the most significant contributors when there are a mass of variables and interactions between variables. The decision tree model finds the strongest predictors by the tree “learning” to split the sample into subsets to improve the predictions. The split will end if there is no improvement in prediction anymore.

All the analyses were conducted by SPSS 25.0. Based on previous experiences, the subgroup sample size should be at least 5% of the entire sample to avoid the model’s instability (18). Therefore, by following the criteria, for the normally developed group, the minimal subgroup sample size was set at 444, and for the chronic-condition group, the smallest sample size was set at 154. The current study applied 10-fold cross-validation procedures. The average risk estimation was obtained from the ten random sub-datasets.

## Results

The descriptive statistics (Table 1) showed adolescents in both groups reported higher than the median point of the emotional health measurement, which indicated a relative health condition. Compared to the normally developed adolescents, the emotional health of chronic-condition(s) adolescents was poorer (*t* = −11.037, *p* < 0.01, Cohens’ d = −0.233).

**Table 1:** The descriptive statistics of sample characteristics.

Normally developed group	N	Mean	Std. Dev	Min	Max
Emotional health	8,381	3.567	1.006	1.000	5.000
Male	4,532				
Female	4,342				
11-year-old-grade	2,874				
13-year-old-grade	3,097				
15-year-old-grade	2,903				
FAS	8,678	2.339	0.391	1.000	3.170
Subjective wealth	8,802	3.970	0.806	1.000	5.000
Talks to parents	8,668	3.913	0.875	1.000	5.000
Family support	8,692	5.042	2.263	1.000	7.000
School-related support	8,636	4.307	0.695	1.000	5.000
Parental monitoring	8,371	3.428	0.549	1.000	4.000
Family activity	8,098	2.805	0.728	1.000	5.000
**Chronic group**	**N**	**Mean**	**Std. Dev**	**Min**	**Max**
Emotional health	2,929	3.325	1.063	1.000	5.000
Male	1,515				
Female	1,552				
11-year-old-grade	765				
13-year-old-grade	1,104				
15-year-old-grade	1,198				
FAS	2,996	2.349	0.403	1.000	3.170
Subjective wealth	3,042	3.890	0.865	1.000	5.000
Talks to parents	2,994	3.760	0.907	1.000	5.000
Family support	2,998	5.058	2.14	1.000	7.000
School-related support	2,979	4.224	0.769	1.000	5.000
Parental monitoring	2,892	3.356	0.578	1.000	4.000
Family activity	2,809	2.364	0.755	1.000	5.000

### CRT for normally developed adolescents

**Figure 1:**
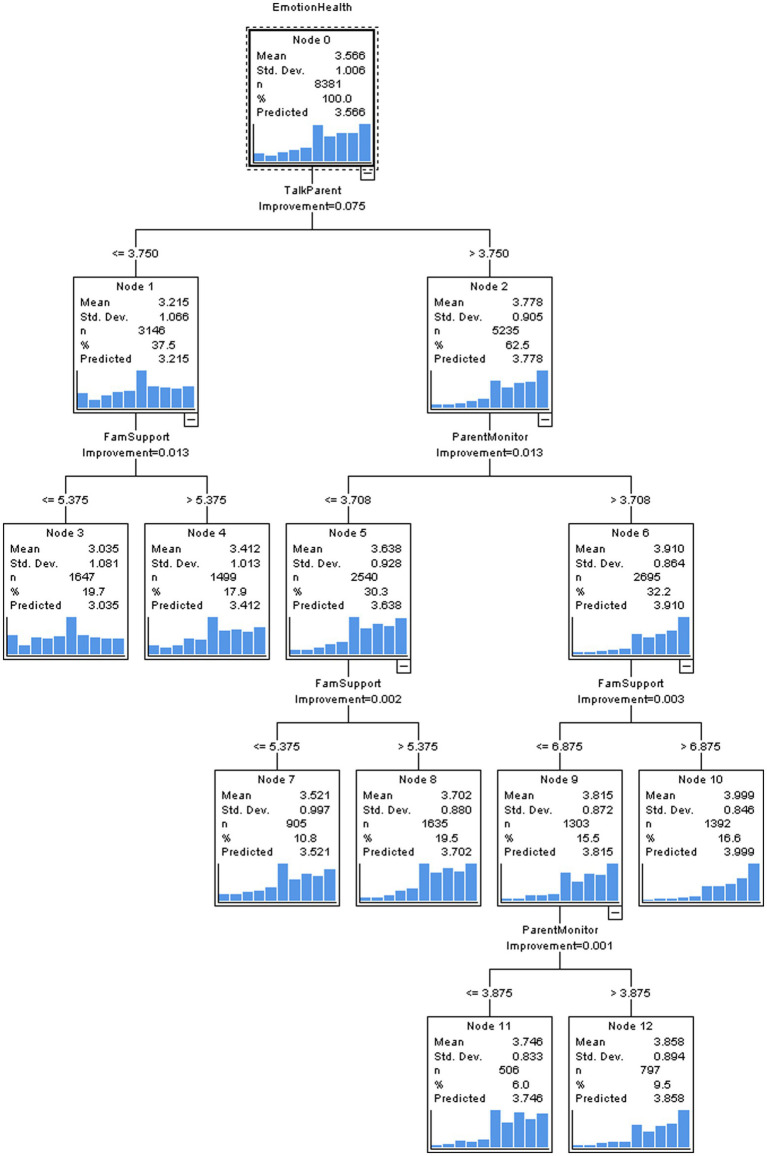
The CRT model to predict normally developed adolescents’ emotional health by family-related factors.

There was not a marked difference in risk between the entire-sample-based estimation and cross-validation estimation (0.908 vs. 0.913), which indicated good stability and validity of the CRT model. This model selected three important predictors for adolescents’ emotional symptoms: talks to parents, parental monitoring, and family support (see Figure 1). The first node was split by “talks to parents” and the cut-off value was 3.750, which meant adolescents with “talks to parents” scores over 3.750 demonstrated less tendency to develop emotional symptoms. For participants with “talks to parents” scores below 3.750, the next split node was decided by “family support.” Among them, adolescents with “family support” scores less than 5.375 faced higher risk of emotional symptoms. Among adolescents with “talks to parents” scores higher than 3.750, the next key node was split by “parental monitoring.” The results suggested parental monitoring was a protective factor for adolescents’ emotional health. The subsequent split after “parental monitoring” was “family support” again. Likewise, adolescents who perceived less family support experienced more frequent emotional symptoms. The whole model explained 10% variance of emotional health.

The decision tree model considered the interactions between predictors when selecting the vital contributors. For deeper exploration, the standardized importance analysis was applied to focus on the importance of each family factor without consideration of the interactions. The results suggested “talks to parents” was the most critical determinant of adolescents’ emotional health. The subsequent decisive family factors in order were parental monitoring and family support (see the Supplementary Figure S1).

### CRT for adolescents with chronic condition(s)

**Figure 2:**
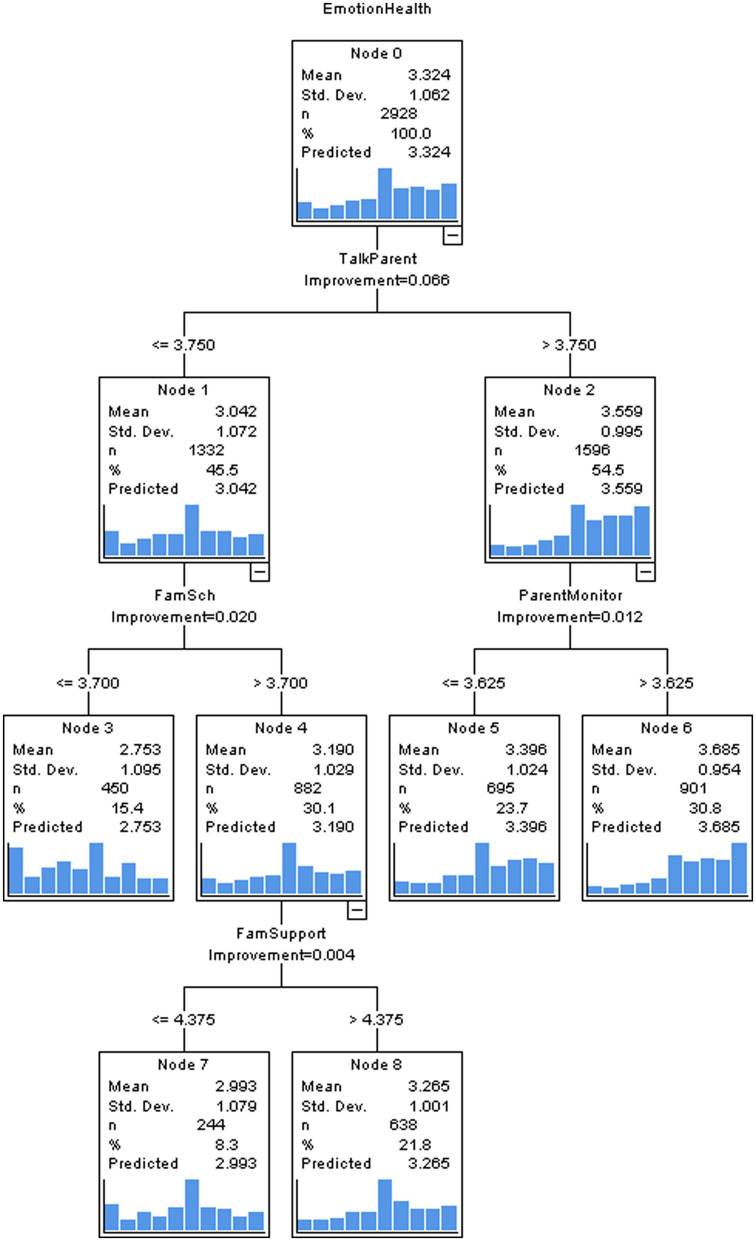
The CRT model to predict adolescents with chronic condition(s) by family-related factors.

The CRT model for adolescents with chronic condition(s) was stable and validated because the risk estimation based on the entire sample (1.027) and that based on cross-validation (1.053) were the same. The CRT model (see Figure 2) elected “talks to parents,” “school-related family support,” “parental monitoring,” and “family support” as significant family contributors to chronic-diseases adolescents’ emotional health. The key characteristic splitting the first node was “talks to parents” and the cut value was 3.750. Adolescents with “talks to parents” scores higher than 3.750 were next divided by “parental monitoring.” More parental monitoring decreased the tendency of emotional symptoms. Among adolescents with “talks to parents” scores lower than 3.750, the subsequent split was “school-related family support.” There was no split again if “school-related family support” scores were below 3.700. When “school-related family support” exceeded 3.700, the next split was determined by “family support,” and the cut-off value was 4.375. “family support” promoted ill adolescents’ emotional health. The CRT model suggested the four family factors accounted for 9% variance of emotional health.

The normalized importance of each family contributor demonstrated that among adolescents with chronic condition(s), for preventing emotional problems, the importance order of family factors was “talks to parents,” followed by “parental monitoring,” “family support,” and “school-related family support” (see the Supplementary Figure S2).

## Discussion

The results reveal that for normally developed adolescents, there are three key family influencers on emotional health: talks to parents, parental monitoring, and family support. And, except for the mentioned three family environmental factors, adolescents with chronic conditions additionally benefit from school-related family support.

The finding that adolescents’ talks to parents can predict emotional health is consistent with previous findings. A theory argued that open parent-adolescent communication encourages adolescents to express feelings and concerns, and at the same time, it offers parents teaching opportunities to shape adolescents’ positive coping strategies. Therefore, parent-adolescent talks are conducive to adolescents’ mental health (19). Except for our finding, a Canadian study also provided evidence that parent-adolescent communication directly improves adolescents’ wellbeing (20). Moreover, a longitudinal investigation indicated that parent-adolescent communication buffers the negative tendency of adolescents’ emotional health (21).

The results show that parental monitoring is another important contributor to adolescents’ emotional health, and this conclusion corroborates with previous studies. Bacchini and his colleagues suggested that parental monitoring minimizes the negative effect of violence environmental on adolescents’ mental health and behavioural development (22). And it is worth noting that the effect of parental monitoring is highly associated with parent-adolescent communication. According to Stattin and Kerr’s theory, parental monitoring does not play the role alone because if adolescents refuse to disclose their daily activities and concerns, parental monitoring cannot be effective (23). In fact, an empirical study found that adolescents’ talks with parents lead to a better emotional health outcome through parental monitoring (24). CRT model is a non-linear model and demonstrates the interactions between independent variables. For both normally developed and chronic-ill adolescents, parental monitoring is a characteristic after the split “talks to parents,” which means parental monitoring shows a positive effect on emotional health by interacting with parent-adolescent talks. Thus, our finding also echoes the theory.

Family support is proven by our CRT models as another protective factor. As one of the social supports for adolescents, family support impacts mental and behavioural development (25, 26). The stress-buffering model pointed out that social support buffers the negative effect of daily stressful events on mental health (27). Besides, as suggested by a meta-analysis, overall social support is associated with adolescents’ wellbeing, and specifically, family support is an important type of social support for adolescents (28). Moreover, an empirical study found that family support decreases the possibility of internal disorders in adolescents who are victims of cyberbullying (29).

On the basis of the findings, compared to normally developed adolescents, those with chronic disease or disability need more parental support in school-related activities to maintain emotional health. The reason might be chronic-disease/disability adolescents face more challenges in school settings, for instance, frequent absence due to hospitalization, falling behind the school work, and social rejection from healthy peers (30). For overcoming these challenges, school-related support is necessary. To my best knowledge, there was no related evidence yet in terms of the effect of parental school-related support on ill adolescents’ mental health. However, researchers summarized that in school settings, direct supportive practice improves chronic-disease adolescents’ academic performance, life quality, and wellbeing (31). Our findings extend the conclusion from direct school support to parental school-related support. Thus, I suggest further researchers investigate the moderator role of adolescents’ health condition in the relationship between parental school-related support and mental health.

I acknowledge this study is limited by the use of secondary data, which means researchers cannot control the data quality. Besides, the HBSC database only focused on early-stage adolescents, which meant the late adolescents were missing in the current study. However, the HBSC database strictly follows the two-stage sampling method, and it has been used in articles published in high-impacted international journals. The current study also shows that the database provides mass valuable information.

## Conclusion

Compared to the normally developed adolescents, chronic- disease/disability adolescents face more risks to emotional health. The current study suggests that talks with parents, family support, and parental monitoring are protective factors for all adolescents’ emotional health. For adolescents with chronic condition(s), there is one more significant family environmental factor contributing the emotional health, school-related parental support. Therefore, this study advocates intervention programmes to enhance parent-school communication and cooperation to promote chronic-ill adolescents’ emotional health.

## Data availability statement

Publicly available datasets were analyzed in this study. This data can be found at: https://hbsc.org/data.

## Ethics statement

The studies involving human participants were reviewed and approved by The Institutional Research Ethics Committee of the Faculty of Physical Culture, Palacky University, Olomouc, approved the data collection on 04 March 2016, No. 9/2016. Written informed consent to participate in this study was provided by the participants’ legal guardian/next of kin.

## Author contributions

The author confirms being the sole contributor of this work and has approved it for publication.

## Conflict of interest

The author declares that the research was conducted in the absence of any commercial or financial relationships that could be construed as a potential conflict of interest.

## Publisher’s note

All claims expressed in this article are solely those of the authors and do not necessarily represent those of their affiliated organizations, or those of the publisher, the editors and the reviewers. Any product that may be evaluated in this article, or claim that may be made by its manufacturer, is not guaranteed or endorsed by the publisher.

## Footnotes

^1^ www.hbsc.org
