# Supplementary figures and images for: Family factors to predict adolescents’ emotional health by decision tree model: A comparison between normally developed group and chronic-condition group
Source: Front Public Health. 2023 Mar 16;11:1087547. doi: 10.3389/fpubh.2023.1087547 (PMC10060630; doi:10.3389/fpubh.2023.1087547)

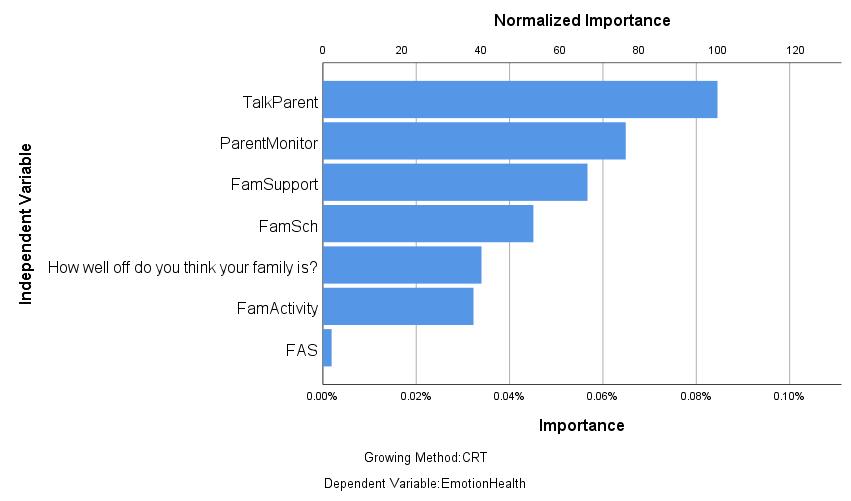

Supplement: Supplementary file 1 [file Image_1.JPEG]

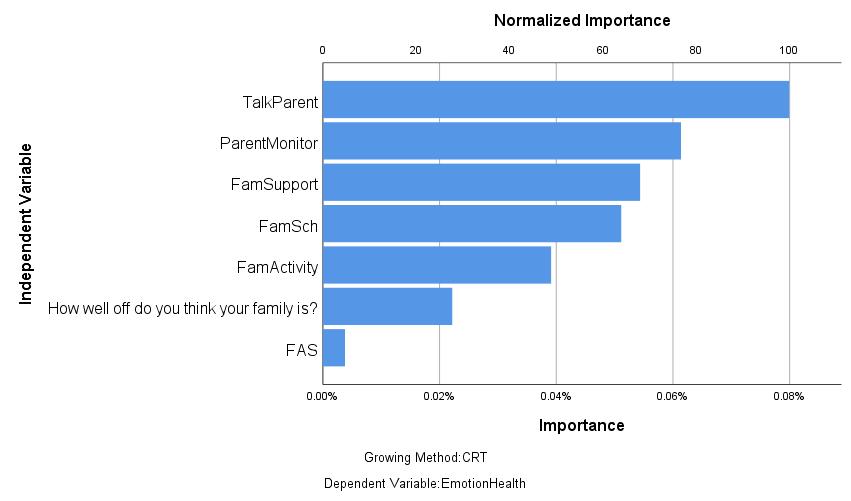

Supplement: Supplementary file 2 [file Image_2.JPEG]
